# Comment on: The use of anterior subcutaneous internal fixation (INFIX) for treatment of pelvic ring injuries in major trauma patients, complications and outcomes

**DOI:** 10.1051/sicotj/2019033

**Published:** 2019-10-18

**Authors:** Rahul Vaidya

**Affiliations:** Department of Orthopaedic Surgery, The DMC Heart Hospital 311 Mack Avenue – 5th floor Detroit MI 48201 USA

In regard to a recent paper published in your journal Sicot J: The use of anterior subcutaneous internal fixation (INFIX) for treatment of pelvic ring injuries in major trauma patients, complications and outcomes by Richard Steer, Ganesh Balendra, Justin Matthews, Martin Wullschleger, and James Reidy.

We have an issue with the introduction of the article on the origins of the INFIX technique. The authors state

“The earliest description of an INFIX was published in the German literature in 2009 [9] with a subsequent mid-term follow-up cohort from the same authors in the English literature in 2013 [8]” [1].

The problem of anterior external fixation was recognized by many groups and pelvic surgeons over the last 50 years. Three separate innovations were developed to address this issue in publications and patents from 2007 to 2012 (and we are certain many more will follow). The “Pelvic Bridge” was developed with plates that connected the iliac crest to the symphysis lying above the external oblique fascia and inguinal ring. It was originally formed with two plates and screws and modified to two-plate rod constructs by Parsell DE, Cole PA in 2007 [2, 3] (Figure 1). The “INFIX” is a construct that uses supraacetabular pedicle screws placed at or above the Anterior Inferior Iliac Spine with a rod that sits in the subcutaneous space in an area called the “bikini line” by Vaidya R in 2008 [4–6] (Figure 2). The Crossover Pelvic Internal Fixator is a construct using Pedicle screws on the inner table of the Ilium with rods similar to the Pelvic Bridge which travel to the area of the symphysis pubis and could be constructed with 1 rod or 2 described by the German Group Kuttner M, Klaiber A, Lorenz T, Füchtmeier B, Neugebauer R in 2009 [7] (Figure 3). All these techniques are used in conjunction with posterior fixation in unstable pelvic ring injuries and have the ability to distract or compress the anterior injury. Significant thought and work went into these innovations which all address anterior pelvic instability. We commend the other innovators on their work and techniques for which clinical results have already been reported [2, 3, 7, 8].

**Figure 1:**
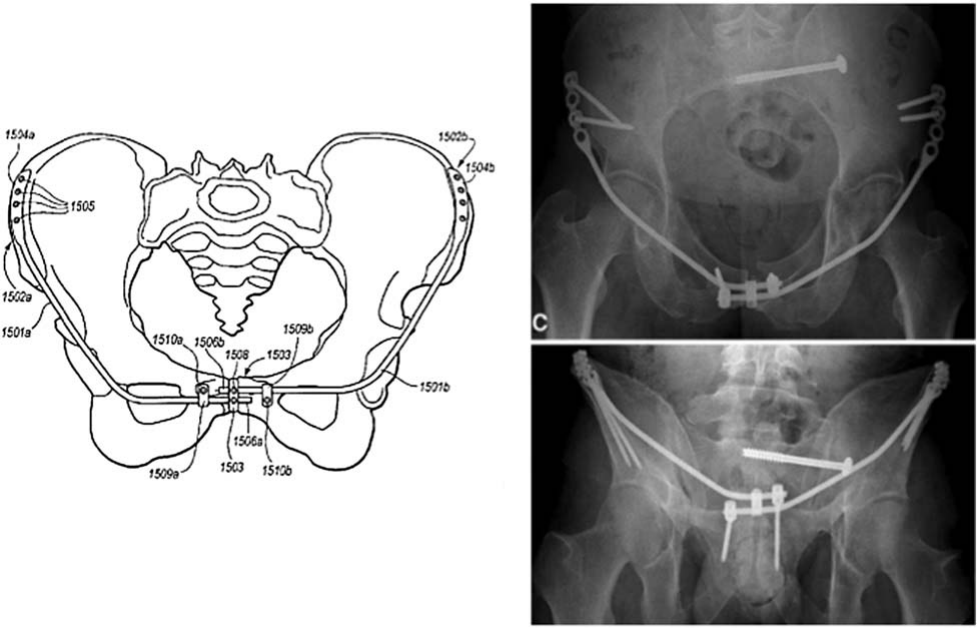
Parsells and Cole (2007) “Pelvic Bridge.”

**Figure 2:**
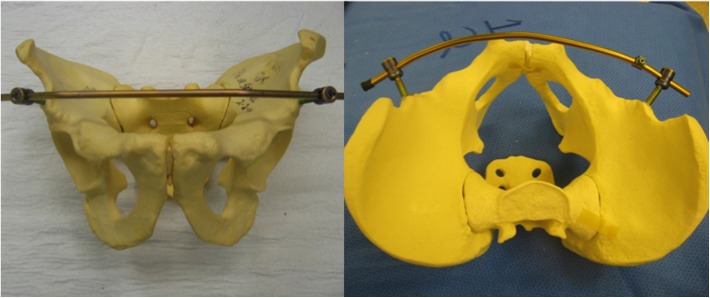
“INFIX” Vaidya (2008).

**Figure 3:**
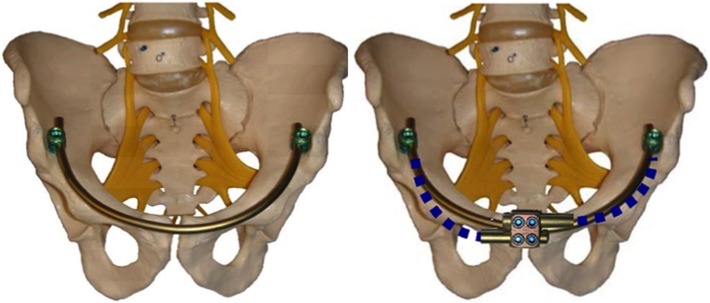
Kuttner et al. (2009) “The Cross-over Pelvic Internal Fixator”.

The technique described in this paper by Steer et al. is the INFIX first described in 2008 by Vaidya [4].

Steer et al. [1] go on to say that the German Group published a clinical report with subsequent midterm follow-up of their technique in 2013 [8]:

“with a subsequent mid-term follow-up cohort from the same authors in the English literature in 2013.”

In 2013 the German Group published a second paper now using the INFIX technique described by Vaidya et al. in 2008 and 2012, having switched their construct now using supraacetabular screws instead of intra pelvic screws and the space noted to be the bikini line instead of the bar coming to the symphysis first described by Cole et al. in 2007 [2, 3]. The reason patents were awarded to Parsells and Cole [2] and Vaidya [4] were that they were the first to describe the respective techniques.

## Conflict of interest

Dr. Vaidya: US patent for anterior subcutaneous internal pelvic fixator. But no conflict of interest with this paper except that mentioned in the letter on the history of INFIX.
